# High-throughput screening of environmental polysaccharide-degrading bacteria using biomass containment and complex insoluble substrates

**DOI:** 10.1007/s00253-020-10469-3

**Published:** 2020-02-29

**Authors:** Estela C. Monge, Marios Levi, Joseph N. Forbin, Mussie D. Legesse, Basil A. Udo, Tagide N. deCarvalho, Jeffrey G. Gardner

**Affiliations:** 1 Department of Biological Sciences, University of Maryland, Baltimore County, Baltimore, MD USA; 2 Keith R. Porter Imaging Facility, University of Maryland, Baltimore County, Baltimore, MD USA

**Keywords:** 3D printing, *Cellvibrio japonicus*, Cellulose, Chitin, Lignocellulose, Polysaccharide

## Abstract

Carbohydrate degradation by microbes plays an important role in global nutrient cycling, human nutrition, and biotechnological applications. Studies that focus on the degradation of complex recalcitrant polysaccharides are challenging because of the insolubility of these substrates as found in their natural contexts. Specifically, current methods to examine carbohydrate-based biomass degradation using bacterial strains or purified enzymes are not compatible with high-throughput screening using complex insoluble materials. In this report, we developed a small 3D printed filter device that fits inside a microplate well that allows for the free movement of bacterial cells, media, and enzymes while containing insoluble biomass. These devices do not interfere with standard microplate readers and can be used for both short- (24–48 h) and long-duration (> 100 h) experiments using complex insoluble substrates. These devices were used to quantitatively screen in a high-throughput manner environmental isolates for their ability to grow using lignocellulose or rice grains as a sole nutrient source. Additionally, we determined that the microplate-based containment devices are compatible with existing enzymatic assays to measure activity against insoluble biomass. Overall, these microplate containment devices provide a platform to study the degradation of complex insoluble materials in a high-throughput manner and have the potential to help uncover ecologically important aspects of bacterial metabolism as well as to accelerate biotechnological innovation.

**Electronic supplementary material:**

The online version of this article (10.1007/s00253-020-10469-3) contains supplementary material, which is available to authorized users.

## Introduction

Bacteria and fungi are integral drivers of the calculated 10^9^-ton annual global carbon cycle (Behrenfeld et al. 2006; Field et al. 1998; Luo et al. 2015; Trivedi et al. 2016). Consequently, substantial efforts have been invested to harness the capabilities of carbohydrate-degrading bacteria for the production of renewable fuels and chemicals (Chandel et al. 2018; Erickson et al. 2012; Mathews et al. 2015). It is the degradative activities of carbohydrate-active enzymes (CAZymes [http://www.cazy.org]) from these microorganisms that are essential for both environmental and industrial carbohydrate degradation (Lombard et al. 2014). While there are many detailed structural and biochemical studies of individual CAZymes using purified carbohydrate substrates, knowledge of the enzymatic activities against unprocessed complex insoluble substrates is not as robust.

There are significant challenges to measuring enzymatic activity using complex insoluble substrates. There is an inherent incompatibility between the aqueous systems that CAZymes need to function and the inability of substrates such as lignocellulose and chitinous biomass to be solvated (Gao et al. 2013; Goacher et al. 2014; Van Dyk and Pletschke 2012; Weiss et al. 2013). Therefore, many studies rely on artificial substrates that have either enhanced solubility or soluble chromogenic dyes to help visualize enzymatic activity. While artificial substrates have been informative using isolated individual enzymes, these types of modified polysaccharides do not provide insight into how CAZymes function as part of a concerted nutrient acquisition response by a microorganism. Additionally, the kinetic parameters of a CAZyme against an artificial substrate do not reflect how an enzyme would behave in either an industrial or an environmental setting (Ivanen et al. 2009). To account for these shortcomings, many studies have used pre-treated complex biomass. For lignocellulose alone, there are a multitude of pre-treatments available, and while several of them are industrially relevant, most have the same shortcomings as artificial substrates in addition to producing side products that can adversely affect enzyme activity (Galbe and Zacchi 2007; Mosier et al. 2005; Ravindran and Jaiswal 2016).

In parallel to characterizing purified CAZymes, there have been several attempts to use authentic complex insoluble substrates to screen for bacterial strains with enhanced polysaccharide degradation functions, albeit with low-throughput methods or requiring substantial secondary screening. For example, a previously reported plate-based screening method used the unconventional solidifying agent acrylamide to reduce the number of erroneously characterized environmental samples when screening for carbohydrate-degrading activities (Gardner et al. 2012). The advantage of using acrylamide over agar for plate-based medium was to eliminate environmental bacteria with the ability to degrade agar and use it as a nutrient source, which is a feature found in some marine bacteria. However, substantial secondary screening was required to validate the bacterial isolates. More recently, the use of 3D printed biomass containment devices (BCDs) was employed for experiments using complex insoluble polysaccharides in liquid culture (Nelson et al. 2016). This method used a small porous capsule to contain unprocessed insoluble substrates but allowed the free movement of bacterial cells, media, and enzymes over the course of growth and polysaccharide degradation. While biomass containment was shown to be compatible with bacterial growth experiments using complex insoluble substrates, the devices were not amenable to high-throughput screening or the small volumes required for enzyme assays.

To accelerate the discovery and characterization of carbohydrate-degrading microorganisms and enzymes, a simple high-throughput method of screening is required. This method must have the flexibility to screen for both novel enzymes and strains but also be compatible with complex insoluble polysaccharides. In this report, we have further developed a method that improves the throughput and the versatility of biomass containment devices. Here we benchmark microplate biomass containment devices (mBCDs) to ascertain their utility with natural insoluble substrates. We found that mBCDs facilitate high-throughput screening of enzymes using complex insoluble biomass and do not need any special accommodations to be compatible with existing protocols. Using a commercially available cellulase, we determined that the mBCDs do not interfere with absorbance measurements and allowed us to quantify enzyme activity on untreated lignocellulose.

Additionally, we used mBCDs to quantitatively determine the carbohydrate degradation capabilities of bacterial isolates from diverse environments (*Atta* sp. colonies or wastewater). We verified that several isolates were able to grow using lignocellulose as a sole nutrient source but also learned that some were able to degrade insoluble starch. The use of mBCDs allowed the environmental isolates to be tested simultaneously across suites of complex insoluble polysaccharide substrates. In summary, this proof-of-concept study provides a new approach for researchers to use complex natural substrates to obtain more physiologically or industrially relevant data when characterizing bacterial strains or enzymes.

## Materials and methods

### Design and fabrication of microplate inserts

**Fig. 1:**
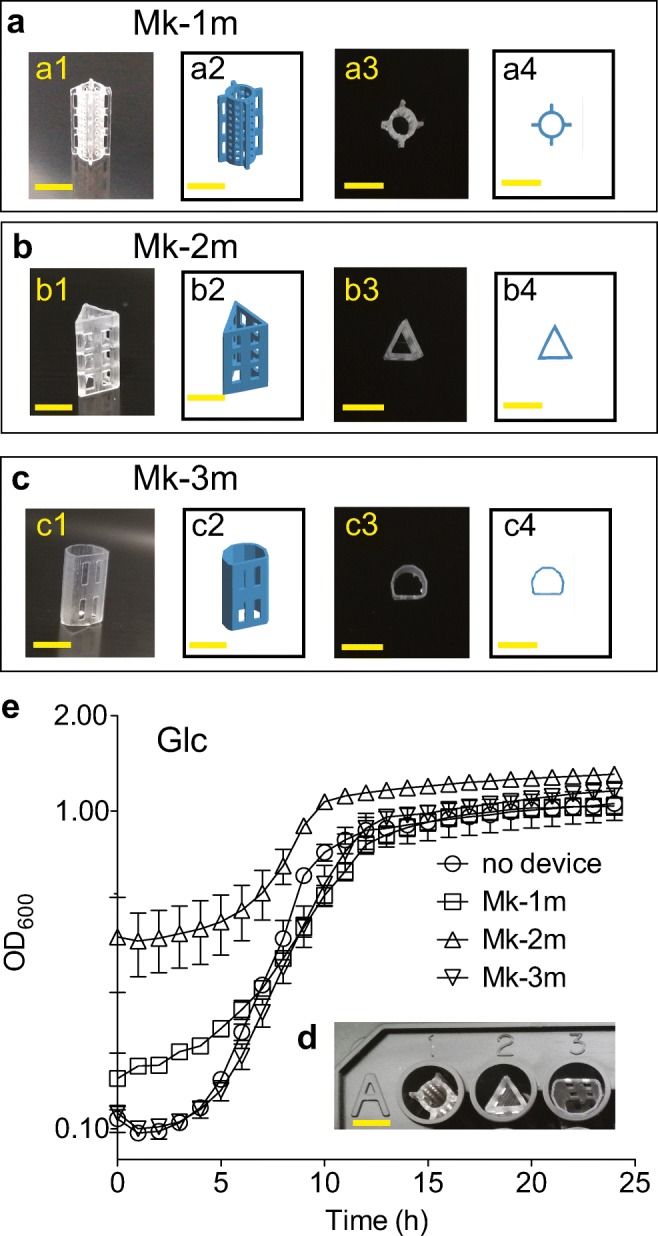
Assessment of three mBCD prototypes for continuous measurements of bacterial growth in a microplate reader. Diagram of Mk-1 m prototype (**a**). Frontal view photograph (a1) and schematic (a2). Top view photograph (a3) and schematic (a4). Diagram of the Mk-2 m prototype (**b**). Frontal view photograph (b1) and schematic (b2). Top view photograph (b3) and schematic (b4). Diagram of the Mk-3 m prototype (**c**). Frontal view photograph (c1) and schematic (c2). Top view photograph (c3) and schematic (c4). In panels (**a–c**), the yellow bar at the bottom of each image corresponds to 5 mm. The inset (**d**) shows the mBCDs placement inside of a single microplate well. Growth analysis of *Cellvibrio japonicus* on minimal media supplemented with 0.5% glucose (Glc) to evaluate growth dynamics (**e**). All experiments were done in biological triplicates using wild-type *C. japonicus* at 30 °C in a microplate with a constant level of aeration. Error bars indicate standard deviation, but at times are too small to be depicted

Microplate biomass containment devices (mBCDs) were designed to fit inside a single well of a standard 96-well plate (Corning #3370; well height: 10 mm; well diameter: 5.5 mm; well wall thickness: 0.5 mm). Three shapes of devices were fabricated for evaluation: Mk-1 m was a porous tube (pore size: 0.5 mm^2^) with perpendicular radial fins (Fig. 1a); Mk-2 m was a triangular pipe with six rectangular pores per face (height: 1.25 mm, width: 1 mm) (Fig 1b); and Mk-3 m was a flattened open cylinder, with the flattened face having four pores (height: 3 mm, width: 1 mm) (Fig. 1c). The mBCDs were designed using the programs OpenSCAD (openscad.org), Meshmixer (meshmixer.com), and PreForm (formlabs.com/tools/preform). Fabrication of the mBCDs used a Form 2 stereolithographic 3D printer (Formlabs) with clear methacrylate resin (RS-F2-GPCL-04) as per manufacturer instructions and cured via irradiation with a UV lamp (36 W UVA lamp; Salon Edge) for 10 min per printed face. Once polymerized, the mBCDs were able to withstand autoclave sterilization (121 °C and 16 PSI for 30 min; gravity cycle) without any adverse effects, which was also previously shown for test tube BCDs (Nelson et al. 2016).

### Bacterial growth conditions

*Cellvibrio japonicus* Ueda107 (NCIMB 10463) and *E. coli* K-12 (NCIMB 10218) were obtained from the National Collections of Industrial, Marine, and Food Bacteria (Aberdeen, Scotland, UK). Several environmentally isolated strains were tested over the course of this work and are available upon request by other researchers, including *Arthrobacter nicotianae* WW33, *Klebsiella oxytoca* WW55, *Enterobacter* spp. WW64, *Bacillus firmus* LZ66, and *Arthrobacter nicotianae* WW33 (Table S1**).** All strains used in these experiments were grown in defined MOPS minimal medium, as done previously (Gardner et al. 2012; Haft et al. 2012). Glucose was used as a soluble control carbon source at 0.5% (w/v). Insoluble carbon sources were used at the following concentrations: 5% (w/v) β-chitin extracted from squid pen (Batch 20140101, France Chitin), 10% (w/v) glutinous rice (*Oryza sativa subsp. japonica*), 5% (w/v) fungal mycelium (*Aspergillus nidulans*), 5% (w/v) mealworm cuticle (*Tenebrio molitor*), 5% (w/v) α-chitin from *Litopenaeus setiferus* (Sigma, Aldrich), 5% (w/v) cellulose blotting paper (#1703932, BioRad), 10% (w/v) crab shells (*Callinectes sapidus*), and 2.5% (w/v) corn stover (*Zea mays*). The β-chitin (Fig. S1a) and rice grains (Fig. S1b) fit into the pocket between the mBCDs and the microplate well without any substrate manipulation. For α-chitin, we sieved the substrate to select for pieces that were 5 mm^2^ (Fig. S1c). The mealworm cuticle, fungal biomass, and filter paper were fragmented into sizes that fit into the pocket as shown in Fig. S2a to S2c. The crab shell and corn stover were prepared as demonstrated in Fig. S3a and S3b. After fragmentation, these substrates were extensively washed with distilled water prior to autoclave sterilization (121 °C and 16 PSI for 30 min; gravity cycle). To prepare a growth experiment, the mBCDs and carbon sources were sterilized separately and then assembled following aseptic technique protocols (Fig. S1a) in a sterile 96-well microtiter assay plate. The mBCD was placed into a well first, and then the insoluble carbon source was placed sterilely into the substrate pocket, followed by addition of a 198-μL defined minimal medium lacking any carbon source. Individual wells were inoculated at a 1:100 dilution from an overnight culture, placing the cells in the large central chamber of the microplate well. Parafilm was used to seal the junction between the lid and the microplate to prevent evaporation during long-term experiments. Short-term growth experiments (24–48-h duration) were analyzed with a TECAN M200Pro or a BioTek EPOCH2 plate reader set to 30 °C with a constant level of high aeration. Long-term growth experiments (> 100-h duration) used a TECAN M200Pro microplate reader for data acquisition but were incubated in an Excella E24 shaker (New Brunswick Scientific) set to 30 °C with a high level of aeration (200 RPM). For both types of experiments, growth was measured using absorbance readings at 600 nm (OD_600_).

### Enzymatic activity assays

For enzymatic assays, 2 mg of filter paper, corn stover, or carboxymethyl cellulose (CMC) were incubated with 200 μL of citrate buffer (pH 5.4) and a commercial endoglucanase from *Aspergillus niger* (E-Cellan, Megazyme) at a concentration of 40 U/mg. One unit of cellulase activity was defined as the amount of enzyme required to release one μmole of glucose-reducing sugar equivalents per min from carboxymethyl cellulose at a concentration of 10 mg/mL in sodium acetate buffer (100 mM), pH 5.4 at 40 °C (Fig S4a). We assessed the degradation of each substrate at 0, 4, 8, and 24 h. Over the course of the experiment samples were taken, flash frozen, and then kept at − 80 °C for further analysis. After thawing all samples, glucose was quantified using a colorimetric glucose oxidase kit (GAGO-20, Sigma-Aldrich) (Fig. S4b) with the following adaptations to a microplate plate: 50 μL of the enzymatic reaction were put into a well followed by the addition of 100 μL of glucose oxidase/peroxidase reagent. The plate was incubated for 30 min at 37 °C. The reaction was stopped by adding 100 μL of 12 N sulfuric acid. Absorbance was obtained using a TECAN M200Pro microplate reader set to read absorbance at 540 nm. A calibration curve was also generated against a known concentration of glucose to determine the amount of glucose released from the experimental samples.

To screen for enzyme activity from environmental samples, *Arthrobacter nicotianae* WW33 was grown in defined MOPS minimal media supplemented with 0.5% (w/v) soluble starch (Fisher Chemical) as the only source of carbon to induce the secretion of starch-degrading enzymes. For the activity screen, the supernatant was collected and filtered through a 0.22-μm Spin-X centrifuge cartridge. The total concentration of protein in the filtrated supernatant was determined by Bradford assay (Bio-Rad Laboratories, Inc., Hercules, CA) using BSA as the standard (Bradford 1976). Next, 250 μL of the filtered supernatant was diluted with defined MOPS minimal medium until a protein concentration between 20 and 30 μg ml^−1^ was reached and then incubated with glutinous rice (10 mg) at 30 °C. Hydrolysis of the rice was assessed at 0, 4, 8, and 24 h. Glucose was then quantified as described above.

## Results

### Assessing utility of microplate biomass containment devices (mBCDs)

During the design and prototyping phase of mBCD fabrication, several parameters were studied to ensure reproducible data acquisition. The core design constraint was to sequester the insoluble substrate to allow the light beam of a spectrophotometer to pass unobstructed to the detector in each well of the microtiter plate. Additionally, the devices had to be adequately porous to allow free exchange and mixing of cells, enzymes, and media while keeping the insoluble substrate fixed. Therefore, the initial three mBCDs (Mk-1 m, Mk-2 m, and Mk-3 m) were designed as inserts that fit snuggly inside of an individual well but had one or several pockets where the insoluble biomass was trapped against the wall of the well (Fig. 1d). To determine if these designs interfered with the path of the spectrophotometer light beam, we grew *Cellvibrio japonicus* using glucose as the sole carbon source with and without mBCDs to ascertain the level of interference during absorbance readings and if the devices influenced growth dynamics. The justification for using *C. japonicus* was that it was the strain used in the previously published BCD study and therefore could be used to make direct comparisons between formats (test tube versus microplate) (Nelson et al. 2016).

All three tested mBCDs prototypes allowed for the continuous measurement of bacterial growth with varying degrees of success (Fig. 1e). Without any device in the well, *C. japonicus* had a growth rate of 0.30 ± 0.02 gen h^−1^, and the maximum optical density (OD_600_) achieved was 1.06 ± 0.12. Wells with *C. japonicus* and Mk-1 m devices resulted in a growth rate that was approximately 33% slower than the no-device control wells (0.20 ± 0.01 gen h^−1^). There was a striking difference in the initial OD_600_ between the Mk-1 m design and the no-device control, either due to direct blocking of the light beam by the mBCD or a shadowing effect. We observed this high level of background when testing the Mk-1 m mBCD in media-only control conditions to assess the interference of the devices over time (Fig. S5). Wells that contained the Mk-2 m devices resulted in a growth rate that was also approximately 33% slower than the no device control wells (0.20 ± 0.05 gen h^−1^). The initial OD_600_ for the Mk-2 m devices was 3.6 times higher than the no device treatment. Wells that contained the Mk-3 m devices resulted in a growth rate that was approximately 4% slower than the no device control wells (0.29 ± 0.02 gen h^−1^). The initial OD_600_ for the Mk-3 m devices was similar to the no device control wells. Regardless of the device tested, all mBCDs allowed for *C. japonicus* to reach similar levels of maximum growth (Table S2). As the Mk-3 m design performed the best during these benchmarking experiments, in addition to being the easiest to manipulate and provided the largest pocket to contain biomass, we conducted all subsequent enzyme and bacterial assays using this design.

### Use of mBCDs uncovers novel growth phenotypes at high solids loading

After determining that the Mk-3 m design was suitable for growth assays, we next evaluated the mBCDs for their ability to parse growth defects from *C. japonicus* mutant strains during short-term experiments (24 to 48 h) using insoluble substrates. As the amount of growth medium in each well is small (~ 200 μL), it was important to determine if the mBCDs could facilitate experiments that have solids loading levels relevant to those found in diverse environments and industrial settings. For example, a previous study determined that 0.25% (w/v) of β-chitin was optimal for test tube BCD growth experiments (Monge et al. 2018). While this level of solids loading might reflect the precision of biomass containment provided by the BCDs, it is too low to be relevant in a biotechnology setting where solids loading can be as high as 20% (Cannella and Jorgensen 2014; Hodge et al. 2008; Kristensen et al. 2009). Therefore, we tested the mBCDs over a range of substrate concentrations that would span both environmental and industrial applications. As a benchmark case, we performed these experiments with β-chitin. Being only constrained by the size of the containment pocket in the Mk-3 m design, we determined that mBCDs could accurately monitor bacterial growth over a broad range of substrate concentrations (Fig. S6). From 2.5% to 15% w/v, we observed a 55% increase in the growth rate as the amount of insoluble substrate was increased with a 54% increase on the maximum density achieved (Table S3). For other insoluble substrates (see Materials and Methods), we used a weight-to-volume ratio that provided the largest difference of the maximum growth between the positive and negative controls.

Arguably, one of the most useful *C. japonicus* strains that has been constructed lacks the type II secretion system, encoded by the *gsp* operon, which is essential for the extracellular transport of CAZymes for this bacterium (Gardner and Keating 2010; Nelson and Gardner 2015). Knowing whether secreted CAZymes are required for the degradation of a substrate, as compared to degradation taking place at the cell surface by membrane-anchored enzymes, allows for a more directed search for CAZymes that have essential activities. For example, a previous study reported that a Δ*gsp* strain was completely unable to utilize β-chitin as a sole carbon source. It was subsequently determined that a single secreted CAZyme, Chi18D (Monge et al. 2018), was the cause of the phenotype. Therefore, to test if mBCDs provide a mechanism to rapidly and quantitatively uncover novel phenotypes for *C. japonicus*, we grew the wild-type and the Δ*gsp* mutant strains using complex insoluble substrates under microplate containment conditions.

**Fig. 2:**
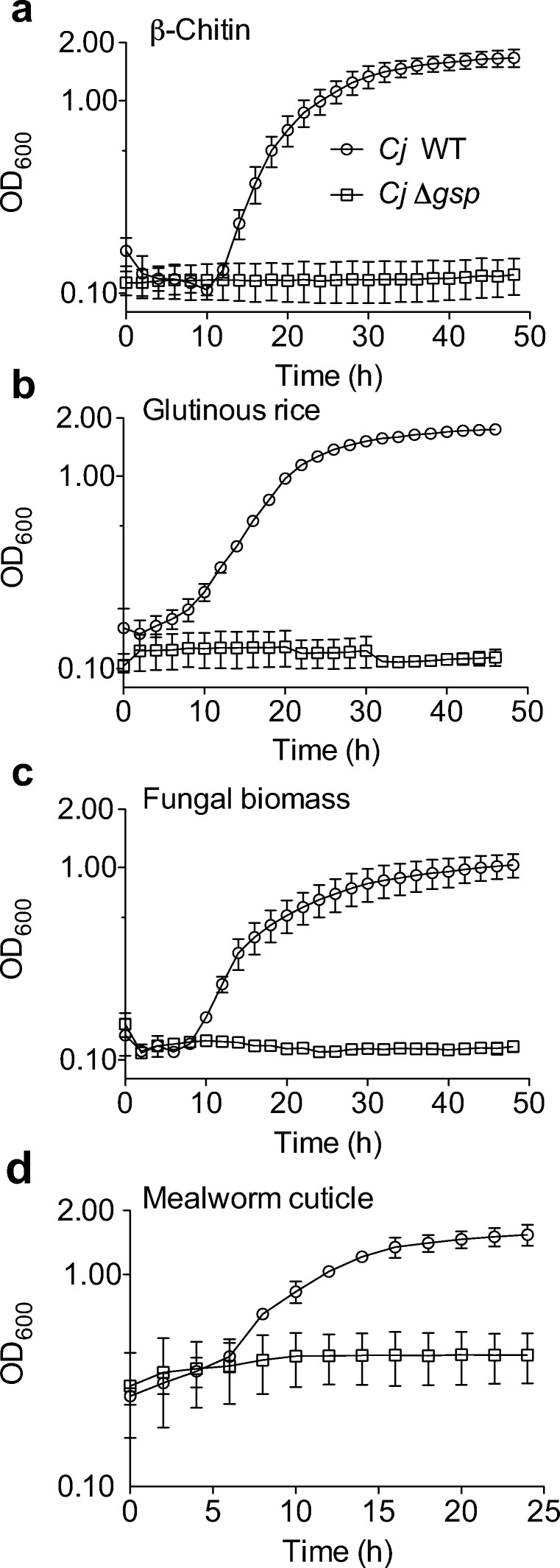
Growth analysis of wild-type *C. japonicus* (circles) and a *Δgsp* deletion mutant (squares) using mBCDs for short-term experiments with insoluble carbon sources. The experiments were performed in a minimal media with (**a**) 5% β-chitin, (**b**) 10% glutinous rice, (**c**) 5% fungal biomass, or (**d**) 5% mealworm cuticle as the only source of carbon. All growth experiments were performed in biological triplicate at 30 °C with high levels of aeration. Error bars indicate standard deviation, but at times are too small to be observed

The data in Fig. 2 shows the growth analysis of the wild-type and the secretion-deficient mutant strain using a series of purified, partially processed, and unprocessed biomasses. Specifically, we used β-chitin as a purified substrate, glutinous rice and mealworm cuticle as semi-processed substrates, and fungal biomass as an unprocessed substrate. For all of the tested substrates, the wild-type was able to grow while the secretion deficient Δ*gsp* mutant did not grow (Fig. 2a to 2d). Interestingly, when using the mealworm cuticle as the sole nutrient source, the initial OD_600_ of the wild-type strain was 1.5-fold higher than the initial OD of the other used substrates (Table S4). Overall, these results provided additional evidence that biomass containment allows for the quantitative measurement of bacterial growth using a diverse array of insoluble substrates.

### Protocol development for long-duration growth experiments using complex insoluble substrates is facilitated with mBCDs

For experiments that last 24–48 h, many commercial plate readers have automated programs that allow for continuous heating, shaking, and absorbance measurements taken at specific intervals. This provides a level of consistency in protocol development and allows for more reproducible data acquisition between replicate experiments. However, *C. japonicus* growth experiments using recalcitrant substrates such as purified α-chitin, crab shells, or filter paper can take over 4 days to reach maximum OD_600_ (Monge et al. 2018; Nelson et al. 2016). These previous experiments used test tube BCDs, which required manual spectrophotometric readings. Therefore, we adapted the short-duration mBCD growth assay protocol and directly compared it to the test tube method to determine if the microplate format provided any benefit to data reproducibility, speed of data generation, or experiment throughput for growth assays using insoluble polysaccharide substrates.

**Fig. 3:**
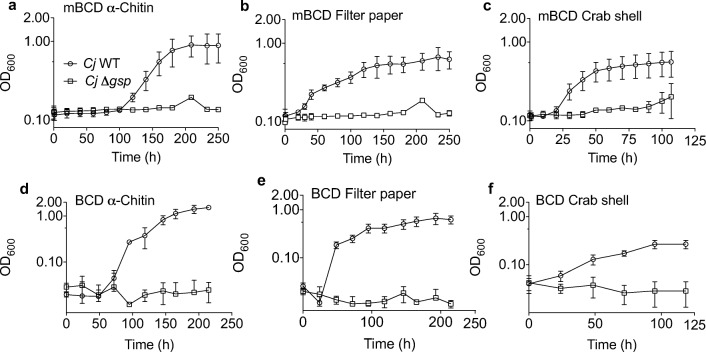
Growth analysis of *C. japonicus* wild-type (circles) and a *Δgsp* deletion mutant (squares) using mBCDs for long-term experiments with insoluble carbon source. The experiments were designed to compare microplate BCDs (**a-c**) with test tube BCDs (D-F). All growth experiments were performed in a minimal media with (**a, d**) 5% α-chitin, (**b, e**) 5% filter paper, or (c, f) 10% crab shell as the only source of carbon. Growth analyses were done at 30 °C with high levels of aeration in biological triplicate. Error bars show standard deviation, but are often too small to be depicted

The degradation of several recalcitrant substrates have been tested using *C. japonicus* and include α-chitin, crab shells, and filter paper (Gardner et al. 2014; Gardner and Keating 2010; Monge et al. 2018). We grew both wild-type and Δ*gsp* mutant strains using these substrates in either a test tube BCD or an mBCD format (Fig. 3). Regardless of format, we observed that wild-type was able to grow and the Δ*gsp* mutant was not able to degrade any of these three substrates, which was similar to those previously described results (Gardner and Keating 2010; Monge et al. 2018; Nelson and Gardner 2015). Using the mBCD format, we were able to save substantial time and run multiple experiments in parallel with a single microplate. Interestingly, we found that the initial OD_600_ readings were higher in the mBCD experiments. While both containment schemes (microplate or test tube) displayed the same growth trends in all tested substrates, the growth rates were lower using mBCDs compared to test tube devices (Table S5), which can be attributed to a reduced level of aeration (Büchs 2001; Duetz et al. 2000). The maximum OD_600_ achieved of the wild-type strain in the long-duration experiments (either format) was lower than the maximum OD_600_ in the short-duration experiments, but this is likely due to wild-type growth not being as robust due to the recalcitrant nature of the substrates. The mBCDs allowed for continuous and automated monitoring of growth experiments in a high-throughput manner despite a reduction of the dynamic range (the difference between the highest and the lowest observed OD_600_) achieved by the wild-type.

### High-throughput environmental isolate screening and phenotype discovery is accelerated with mBCDs

Previous screening approaches using untreated corn stover employed a plate-based method to find lignocellulose-degrading isolates; however, this method was not able to quantitate growth rates and required substantial secondary screening to determine degradation capacity (Gardner et al. 2012). From that study, out of 38 total environmental isolates, 28 were able to utilize all four purified polysaccharide substrates tested, but only four isolates had secreted cellulase activity. These discrepancies were left unexplored, and after the initial screening on untreated lignocellulose, no additional growth analyses were performed with the isolates using complex biomass.

To quantitatively assess insoluble polysaccharide utilization, we reevaluated two Gram-negative and two Gram-positive environmental isolates that were qualitatively ranked as growing the best for all polysaccharide substrates tested. Specifically, we wanted to determine if using the mBCD high-throughput format and complex insoluble substrates would allow us to quantitatively determine which environmental isolates would be the best candidates for further analysis (e.g., genome sequence and/or CAZyme characterization). As expected, all four of the selected environmental isolates were able to use glucose as a sole carbon source; however, what was striking was the wide range of growth rates observed (Fig. S7 and Table S6). Two isolates (WW55 and WW64) had substantially faster growth rates than *C. japonicus* (0.28 ± 0.01 gen h^−1^), and two isolates were markedly slower (WW33 and LZ66). Additionally, the maximum growth attained by the WW33 was slightly higher than *C. japonicus* (0.87 ± 0.01 gen h^−1^), while WW64 was slightly lower. However, all strains were able to achieve similar maximum growth by 24 h, and this is likely the explanation for why all four environmental isolates were scored the same previously in plate assays.

**Fig. 4:**
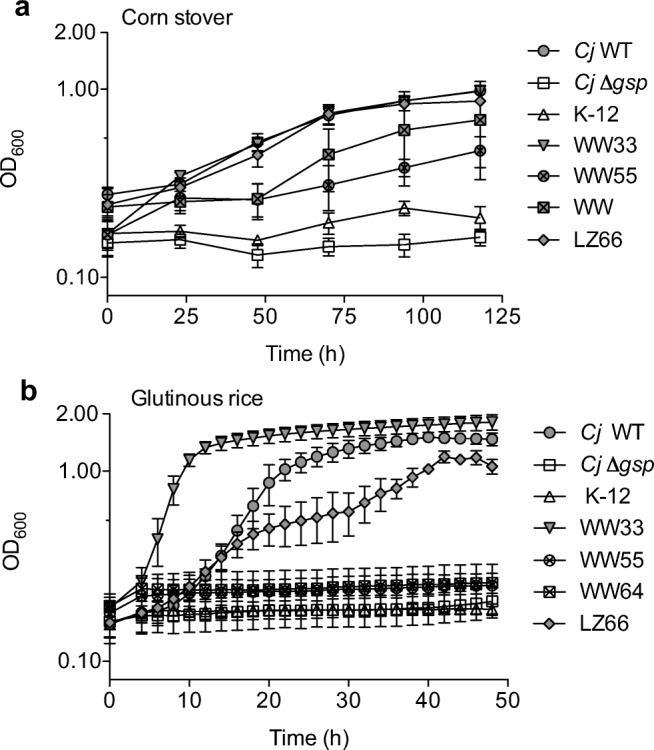
Using mBCDs to screen environmental isolates capacity to degrade insoluble substrates**.** Either (**a**) corn stover or (**b**) glutinous rice was used as the only source of carbon. The tested strains were *Cellvibrio japonicus* (*Cj* WT*), Cellvibrio japonicus* Δ*gsp* (*Cj* Δ*gsp), Escherichia coli* K-12 (K-12), *Arthrobacter nicotianae* (WW33), *Klebsiella oxytoca* (WW55), *Enterobacter* spp. (WW64), and *Bacillus firmus* (LZ66). All growth analysis experiments were done in biological triplicate at 30 °C with high levels of aeration. Error bars indicate standard deviation

Quantitative growth analyses using complex insoluble substrates clearly separated the four environmental isolates based on their degradation capabilities. When the isolates were grown using corn stover as the sole carbon source, the WW33 and LZ66 strains grew similar to wild-type *C. japonicus* both in terms of growth rate (*C. japonicus*, 0.02 ± 0.01 gen h^−1^) and maximum growth (*C. japonicus*, 0.98 ± 0.12 gen h^−1^), while the WW55 and WW64 strains were reduced in both circumstances (Fig. 4a). When glutinous rice was the sole carbon source, there was a clear delineation between strains that could utilize it and those that were completely unable to grow (Fig. 4b). The WW33 isolate had a rapid growth rate compared to *C. japonicus* (WW33, 0.25 ± 0.02 gen h^−^1; *C. japonicus*, 0.13 ± 0.02 gen h^−1^), while the LZ66 strain was slower and displayed a biphasic pattern. The WW64 and WW55 strains were unable to grow using glutinous rice as a substrate (Table S7). These experiments demonstrate the power of quantitative growth analyses using complex insoluble substrates in combination with biomass containment. While beyond the scope of this report, the WW33 isolate is currently undergoing an in-depth genetic and biochemical characterization to determine if any of its CAZymes have potential biotechnology applications.

### Enzyme assays using complex insoluble substrates and mBCDs is streamlined

The primary use of microplate biomass containment was designed to facilitate the measurement of bacterial growth using insoluble substrates; however, we also wanted to determine if mBCDs could streamline existing enzymatic protocols with minimal substrate processing and reduced assay volumes. Many commercially available enzyme activity kits have protocols for high-throughput screening. Therefore, we used mBCDs in combination with carboxymethyl cellulose (CMC), filter paper, or corn stover to assess the compatibility of mBCDs for enzyme activity assays with a high level of solids loading and using a commercial endoglucanase. Using a standard colorimetric glucose oxidase kit, we found that the mBCDs were compatible with microplate format enzymatic assays (Fig. S4). Specifically, over the course of 24 h, the amount of glucose released from a filter paper reaction at 1% solids loading was 5.8 ± 1.4 μg (Table 1). Using corn stover at 1% solids loading the amount of glucose released was 1.4 ± 0.6 μg. When CMC was used as a control substrate, we observed 257.5 ± 42.7 μg of released glucose. This increased amount of released glucose was expected due the soluble nature of the CMC substrate. In all cases, the use of the mBCDs eliminated the need of a centrifugation or filtration step to remove the insoluble substrate as an aqueous sample could be directly taken from the center chamber of the well.

**Table 1:** Enzymatic degradation of CMC, filter paper, and corn stover using mBCDs

	μg of Glc released^a^		
Time (h)	CMC	Filter paper	Corn stover
0	1.8 ± 0.4	0.1 ± 0.1	0.1 ± 0.1
4	47.3 ± 4.9	3.2 ± 1.1	1.5 ± 0.4
8	90.4 ± 8.7	3.6 ± 1.2	1.0 ± 0.7
24	257.5 ± 42.7	5.8 ± 1.4	1.4 ± 0.6

^a^Degradation of each substrate was accessed at 0, 4, 8, and 24 h incubation, and each mean and standard deviation was calculated from biological triplicate experiments. Glucose was quantified as described in the Materials and Methods.

**Fig. 5:**
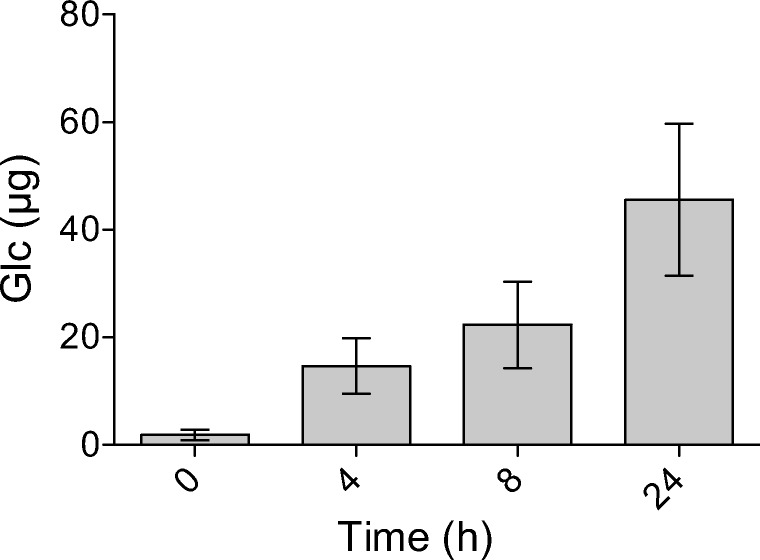
Activity assay of WW33 supernatants against glutinous rice using biomass containment. Glucose released from degradation of the starches in glutinous rice by the hydrolytic activity of enzymes collected from the growth medium of *Arthrobacter nicotianae* (WW33) was determined at 0, 4, 8, and 24 h. Each column represents an average of triplicate measurements, with the error bars depicting standard deviation

To determine if we could employ mBCDs to assay enzyme activity from cell supernatants, we used a microplate-based assay to quantify the hydrolytic activity of the extracellular enzymes from isolate WW33. We then assessed the degradation of glutinous rice contained in an mBCD from the WW33 supernatant at 0, 4, 8, and 24 h incubation. Our activity assay showed an increase in the amount of detected free glucose over the incubation period (Fig. 5). These results indicated that mBCDs could be used for rapid screening of secreted proteins to detect specific types of enzymatic activities.

## Discussion

The strategies used by microbes for efficient bioconversion of recalcitrant polysaccharides have gained interest for ecology and animal health studies along with biotechnological applications. In vivo studies are a useful tool to understand the degradation processes because they characterize physiologically relevant functions (Blake et al. 2018; Gardner and Keating 2010; Monge et al. 2018). However, it is difficult to study bacterial growth or enzyme activity using physiologically relevant substrates because of the insolubility of the substrates. In a previous study, we utilized biomass containment devices (BCDs) to characterize the growth of *Cellvibrio japonicus* with lignocellulose using an 18-mm test tube format (Nelson et al. 2016). In this report, we applied the same principle of biomass containment using a microplate format to assess if this method could be used in a high-throughput manner for cell growth and enzymatic activity measurements.

During the mBCD design phase, the three most influential factors were microplate reader compatibility, experiment duration, and substrate availability. While the default settings of both TECAN M200Pro and BioTek EPOCH2 microplate readers were sufficient to provide reproducible OD_600_ readings with low standard deviation for all three mBCD designed tested, we hypothesize that the Mk-3 m designed worked best because the shape of the device tightly fit inside of the well and left only a 2 mm by 4 mm pocket to hold the biomass against the wall of the well (Fig. 1). This shape prevented erroneous light scattering or shadows that would reduce the accuracy of the reading or create an unacceptably high background, which was observed for the Mk-2 m design (Fig. S5). Furthermore, we speculate that the large central chamber of the Mk-3 m design allowed for better circulation of the media and bacteria while the large side pocket allowed for a relatively high solids loading in a small aqueous volume (15% w/v solids in a 200 μL total volume). Finally, we found that applying parafilm to seal the gap between the lid and microplate mitigated evaporation of media in conjunction with designing the experiments with a perimeter of wells filled with water to prevent evaporation of experimental wells. While this reduced throughput by 37% (60 out of 96 wells available for experimental samples), it did prevent edge effects (Leyva et al. 2008; Lundholt et al. 2003; Pfeifer and Scheel 2009). Using self-adhesive gas-permeable sealing film resulted in lower growth rates and was not suitable for growth assays with mBCDs (Fig. S8).

By using biomass containment, we have begun to uncover the complex mechanisms that *C. japonicus* employs to degrade and consume recalcitrant substrates. This bacterium has over 300 predicted enzymes to degrade and modify polysaccharides, and the locations of these enzymes can be found cytoplasmically, periplasmically, and extracellularly (DeBoy et al. 2008; Gardner 2016). In order to evaluate if secreted enzymes are essential for polysaccharide degradation of a particular substrate, we previously generated a *C. japonicus* deletion mutant that no longer has a type II secretion system, as encoded by the general secretory pathway (*gsp*) operon (Nelson and Gardner 2015). This mutant was able to determine if secreted CAZymes were essential for the utilization of soluble polysaccharides (e.g., required for CMC but dispensable for xylan) (Blake et al. 2018; Gardner and Keating 2010). The current challenge studying this biological process in *C. japonicus* or another saprophytic bacterium is that to obtain ecologically or industrially relevant data complex insoluble substrates need to be used. Prior to biomass containment, there was not a method to measure bacterial growth or substrate degradation in real-time and in a high-throughput manner using complex insoluble substrates. Now we can obtain quantitative growth data in real-time and in a high-throughput manner as shown by our proof-of-concept data for several insoluble substrates (filter paper, corn stover, crab shells, β-chitin, glutinous rice, fungal biomass, and mealworm cuticle).

The substrates tested provided a range of complexities to quantitatively assess *C. japonicus* growth rates (as an approximation of degradation capacity) and ascertain the importance of secreted CAZymes for degradation. When quantitatively comparing the length of lag phase, the generation times, and the maximum growth attained, we found consistently that the long-duration experiments were reduced compared to the short-duration experiments (Table S4 and S5). These results were unsurprising as similar data were shown with test tube BCDs (Nelson et al. 2016). The inability of the Δ*gsp* mutant to grow on complex substrates like rice, fungal biomass, and mealworm cuticle was expected, due to the insoluble nature of these substrates. In the case of glutinous rice, this result was expected because *C. japonicus* has 22 predicted starch-specific CAZymes, and 11 of these are predicted to be secreted. However, only seven of the secreted enzymes are predicted to be GH13 amylases, which would be required to degrade the 30% of insoluble starch in a polished rice grain (Beloshapka et al. 2016; U.S FDA 2018). This suggests that only one or a few CAZymes are essential for the degradation of insoluble starch. The Δ*gsp* mutant was also unable to grow on complex insoluble substrates (fungal biomass and mealworm cuticle) (Fig. 2). This was expected given that fungal biomass is composed of insoluble glucans in addition to chitin, which the bacterium needs to degrade extracellularly. Similarly, mealworm cuticles have lipoproteins and waxes in addition to chitin that could be used as growth substrates (Andersen et al. 1973; Lockey 1988; Song et al. 2018; Wigglesworth 1948). For either substrate, we predicted that secreted enzymes are essential. Subsequent studies are ongoing to tease apart which components of rice, fungal mycelium, or mealworm cuticle are being utilized by *C. japonicus*, and identifying the secreted enzymes that are essential to degrade these complex substrates.

Screening environmental isolates for novel CAZyme activities currently relies heavily on pre-treated, purified, or chemically modified substrates. While many of these methods have helped to characterized CAZymes, often these enzymes do not perform as expected when tested against complex untreated substrates (Jiang et al. 2017; Sharma et al. 2012). A reason for this discrepancy is likely due to the elimination of the native context in which complex polysaccharides are presented. Therefore, environmental screening protocols need to incorporate authentic biomass to be more effective. While previous attempts to use untreated substrates were able to obtain environmental isolates, the use of plate-based screening only gave qualitative results (Gardner et al. 2012). The reevaluation of the “top-performing” strains from this report using quantitative growth analyses with biomass containment made clear distinctions between the isolates (Fig. 4). Given that the isolates were obtained from a leaf-cutting ant colony or wastewater, the two most ecologically recalcitrant substrates to test were lignocellulose and insoluble starch. Similar final OD_600_ readings of the four isolates corroborated the results from the previous study; however, the growth rate measurements clearly indicate that the WW33 and LZ66 strains grow more robustly on corn stover compared to WW55 and WW64. The WW33 and LZ66 isolates, previously identified as *Arthrobacter nicotianae* and *Bacillus firmus*, respectively, were collected from a leaf cutting ant colony, and these two bacterial species have also been found together in the hindguts of giant crane flies (Cook et al. 2007). These two isolates were able to grow using glutinous rice with the WW33 strain significantly outperformed *C. japonicus* in terms of growth rate, which suggests this *A*. *nicotianae* environmental isolate might have industrial potential. Overall, we conclude that quantitative measurements of growth rate are more informative to assess the capabilities of environmental isolates. Future applications of biomass containment approaches could be to generate a detailed study of nutrient succession from leaf litter decomposition in forest or agricultural environments.

Current CAZyme research has focused in the isolation and characterization of novel enzymes (Attia et al. 2016; Iacono et al. 2019; Liu et al. 2017; Munoz-Munoz et al. 2017). While these studies have elucidated the enzymatic mechanisms of several CAZymes using artificial substrates, there are fewer studies that employ environmentally relevant substrates because of the practical difficulties of performing enzymatic assays using these materials. Therefore, we also wanted to assess if mBCDs could simplify enzyme assay experiments that used insoluble substrates, which previously required complex experimental designs (Chundawat et al. 2008; Knutsen and Davis 2002; Qi et al. 2011). While some current methods have high-throughput capability, these protocols have many drawbacks in terms of what substrates can be used, and often require chemical modification. In contrast, the use of mBCDs facilitated enzyme assays using complex insoluble substrates. We found that the tested commercial endoglucanase was more active against carboxymethyl cellulose than against filter paper or corn stover (Table 1). These results are easily explained as a consequence of CMC having increased enzymatic digestibility due to its solubility. While the amount of glucose released from the filter paper and corn stover was considerably lower, biomass containment allowed for the reproducible and quantitative measurement of glucose released from these substrates. Moreover, the mBCDs were a useful tool to aid in the discovery of new microbial enzymes for improving biomass conversion processes. Our supernatant activity assays identified enzyme activity that was secreted from an environmental isolate, which we were able to evaluate in a high-throughput manner using a natural substrate (Fig. 5). In the future, microplate biomass containment could be used to determine enzyme activity for purified proteins, supernatants, or cell-free extracts in conjunction with complex insoluble substrates.

## Electronic supplementary material

ESM 1 (PDF 4590 kb)

## References

[CR1] Andersen SO, Chase AM, Willis JH (1973). The amino-acid composition of cuticles from *Tenebrio molitor* with special reference to the action of juvenile hormone. Insect Biochem.

[CR2] Attia M, Stepper J, Davies GJ, Brumer H (2016). Functional and structural characterization of a potent GH74 endo-xyloglucanase from the soil saprophyte *Cellvibrio japonicus* unravels the first step of xyloglucan degradation. FEBS J.

[CR3] Behrenfeld MJ, O'Malley RT, Siegel DA, McClain CR, Sarmiento JL, Feldman GC, Milligan AJ, Falkowski PG, Letelier RM, Boss ES (2006). Climate-driven trends in contemporary ocean productivity. Nature.

[CR4] Beloshapka AN, Buff PR, Fahey GC, Swanson KS (2016). Compositional analysis of whole grains, processed grains, grain co-products, and other carbohydrate sources with applicability to pet animal nutrition. Foods.

[CR5] Blake AD, Beri NR, Guttman HS, Cheng R, Gardner JG (2018). The complex physiology of *Cellvibrio japonicus* xylan degradation relies on a single cytoplasmic beta-xylosidase for xylo-oligosaccharide utilization. Mol Microbiol.

[CR6] Bradford MM (1976). A rapid and sensitive method for the quantitation of microgram quantities of protein utilizing the principle of protein-dye binding. Anal Biochem.

[CR7] Büchs J (2001). Introduction to advantages and problems of shaken cultures. Biochem Eng J.

[CR8] Cannella D, Jorgensen H (2014). Do new cellulolytic enzyme preparations affect the industrial strategies for high solids lignocellulosic ethanol production?. Biotechnol Bioeng.

[CR9] Chandel AK, Garlapati VK, Singh AK, Antunes FAF, da Silva SS (2018). The path forward for lignocellulose biorefineries: bottlenecks, solutions, and perspective on commercialization. Bioresour Technol.

[CR10] Chundawat SP, Balan V, Dale BE (2008). High-throughput microplate technique for enzymatic hydrolysis of lignocellulosic biomass. Biotechnol Bioeng.

[CR11] Cook DM, DeCrescenzo HE, Upchurch R, Peterson JB (2007). Isolation of polymer-degrading bacteria and characterization of the hindgut bacterial community from the detritus-feeding larvae of *Tipula abdominalis* (Diptera: Tipulidae). Appl Environ Microbiol.

[CR12] DeBoy RT, Mongodin EF, Fouts DE, Tailford LE, Khouri H, Emerson JB, Mohamoud Y, Watkins K, Henrissat B, Gilbert HJ, Nelson KE (2008). Insights into plant cell wall degradation from the genome sequence of the soil bacterium *Cellvibrio japonicus*. J Bacteriol.

[CR13] Duetz WA, Ruedi L, Hermann R, O'Connor K, Buchs J, Witholt B (2000). Methods for intense aeration, growth, storage, and replication of bacterial strains in microtiter plates. Appl Environ Microbiol.

[CR14] Erickson B, Nelson J, Winters P (2012). Perspective on opportunities in industrial biotechnology in renewable chemicals. Biotechnol J.

[CR15] Field CB, Behrenfeld MJ, Randerson JT, Falkowski P (1998). Primary production of the biosphere: integrating terrestrial and oceanic components. Science.

[CR16] Galbe M, Zacchi G (2007). Pretreatment of lignocellulosic materials for efficient bioethanol production. Adv Biochem Eng Biotechnol.

[CR17] Gao D, Chundawat SP, Sethi A, Balan V, Gnanakaran S, Dale BE (2013). Increased enzyme binding to substrate is not necessary for more efficient cellulose hydrolysis. Proc Natl Acad Sci U S A.

[CR18] Gardner JG (2016). Polysaccharide degradation systems of the saprophytic bacterium *Cellvibrio japonicus*. World J Microbiol Biotechnol.

[CR19] Gardner JG, Keating DH (2010). Requirement of the type II secretion system for utilization of cellulosic substrates by *Cellvibrio japonicus*. Appl Environ Microbiol.

[CR20] Gardner JG, Zeitler LA, Wigstrom WJ, Engel KC, Keating DH (2012). A high-throughput solid phase screening method for identification of lignocellulose-degrading bacteria from environmental isolates. Biotechnol Lett.

[CR21] Gardner JG, Crouch L, Labourel A, Forsberg Z, Bukhman YV, Vaaje-Kolstad G, Gilbert HJ, Keating DH (2014). Systems biology defines the biological significance of redox-active proteins during cellulose degradation in an aerobic bacterium. Mol Microbiol.

[CR22] Goacher RE, Selig MJ, Master ER (2014). Advancing lignocellulose bioconversion through direct assessment of enzyme action on insoluble substrates. Curr Opin Biotechnol.

[CR23] Haft RJ, Gardner JG, Keating DH (2012). Quantitative colorimetric measurement of cellulose degradation under microbial culture conditions. Appl Microbiol Biotechnol.

[CR24] Hodge DB, Karim MN, Schell DJ, McMillan JD (2008). Soluble and insoluble solids contributions to high-solids enzymatic hydrolysis of lignocellulose. Bioresour Technol.

[CR25] Iacono RSA, Maurelli L, Curci N, Casillo A, Corsaro MM, Moracci M, Cobucci-Ponzano B (2019). GlcNAc de-N-acetylase from the hyperthermophilic archaeon *Sulfolobus solfataricus*. Appl Environ Microbiol.

[CR26] Ivanen DR, Rongjina NL, Shishlyannikov SM, Litviakova GI, Isaeva-Ivanova LS, Shabalin KA, Kulminskaya AA (2009). Novel precipitated fluorescent substrates for the screening of cellulolytic microorganisms. J Microbiol Methods.

[CR27] Jiang Z, Han B, Liu W, Peng Y (2017). Evaluation on biological compatibility of carboxymethyl chitosan as biomaterials for antitumor drug delivery. J Biomater Appl.

[CR28] Knutsen JS, Davis RH (2002). Combined sedimentation and filtration process for cellulase recovery during hydrolysis of lignocellulosic biomass. Appl Biochem Biotechnol.

[CR29] Kristensen JB, Felby C, Jorgensen H (2009). Yield-determining factors in high-solids enzymatic hydrolysis of lignocellulose. Biotechnol Biofuels.

[CR30] Leyva A, Quintana A, Sanchez M, Rodriguez EN, Cremata J, Sanchez JC (2008). Rapid and sensitive anthrone-sulfuric acid assay in microplate format to quantify carbohydrate in biopharmaceutical products: method development and validation. Biologicals.

[CR31] Liu Z, Gay LM, Tuveng TR, Agger JW, Westereng B, Mathiesen G, Horn SJ, Vaaje-Kolstad G, van Aalten DMF, Eijsink VGH (2017). Structure and function of a broad-specificity chitin deacetylase from *Aspergillus nidulans* FGSC A4. Sci Rep.

[CR32] Lockey KH (1988). Lipids of the insect cuticle: origin, composition and function. Comp Biochem Physiol B: Comp Biochem.

[CR33] Lombard V, Golaconda Ramulu H, Drula E, Coutinho PM, Henrissat B (2014). The carbohydrate-active enzymes database (CAZy) in 2013. Nucleic Acids Res.

[CR34] Lundholt BK, Scudder KM, Pagliaro L (2003). A simple technique for reducing edge effect in cell-based assays. J Biomol Screen.

[CR35] Luo Y, Keenan TF, Smith M (2015). Predictability of the terrestrial carbon cycle. Glob Chang Biol.

[CR36] Mathews SL, Pawlak J, Grunden AM (2015). Bacterial biodegradation and bioconversion of industrial lignocellulosic streams. Appl Microbiol Biotechnol.

[CR37] Monge EC, Tuveng TR, Vaaje-Kolstad G, Eijsink VGH, Gardner JG (2018). Systems analysis of the glycoside hydrolase family 18 enzymes from *Cellvibrio japonicus* characterizes essential chitin degradation functions. J Biol Chem.

[CR38] Mosier N, Wyman C, Dale B, Elander R, Lee YY, Holtzapple M, Ladisch M (2005). Features of promising technologies for pretreatment of lignocellulosic biomass. Bioresour Technol.

[CR39] Munoz-Munoz J, Cartmell A, Terrapon N, Basle A, Henrissat B, Gilbert HJ (2017). An evolutionarily distinct family of polysaccharide lyases removes rhamnose capping of complex arabinogalactan proteins. J Biol Chem.

[CR40] Nelson CE, Gardner GJ (2015). In-frame deletions allow functional characterization of complex cellulose degradation phenotypes in *Cellvibrio japonicus*. Appl Environ Microbiol.

[CR41] Nelson CE, Beri NR, Gardner JG (2016). Custom fabrication of biomass containment devices using 3-D printing enables bacterial growth analyses with complex insoluble substrates. J Microbiol Methods.

[CR42] Pfeifer MJ, Scheel G (2009). Long-term storage of compound solutions for high-throughput screening by using a novel 1536-well microplate. J Biomol Screen.

[CR43] Qi B, Chen X, Su Y, Wan Y (2011). Enzyme adsorption and recycling during hydrolysis of wheat straw lignocellulose. Bioresour Technol.

[CR44] Ravindran R, Jaiswal AK (2016). A comprehensive review on pre-treatment strategy for lignocellulosic food industry waste: challenges and opportunities. Bioresour Technol.

[CR45] Sharma N, Kaushal R, Gupta R, Kumar S (2012). A biodegradation study of forest biomass by *Aspergillus nige*r F7: correlation between enzymatic activity, hydrolytic percentage and biodegradation index. Braz J Microbiol.

[CR46] Song YS, Kim MW, Moon C, Seo DJ, Han YS, Jo YH, Noh MY, Park YK, Kim SA, Kim YW, Jung WJ (2018). Extraction of chitin and chitosan from larval exuvium and whole body of edible mealworm, *Tenebrio molitor*. Entomol Res.

[CR47] Trivedi P, Delgado-Baquerizo M, Trivedi C, Hu H, Anderson IC, Jeffries TC, Zhou J, Singh BK (2016). Microbial regulation of the soil carbon cycle: evidence from gene-enzyme relationships. ISME J.

[CR48] U.S FDA (2018) Food composition databases show foods -- rice, white, long-grain, regular, unenriched, cooked without salt. U.S. Department of Agriculture. https://ndb.nal.usda.gov/ndb/foods/show/20445 Accessed 08 August 2019

[CR49] Van Dyk JS, Pletschke BI (2012). A review of lignocellulose bioconversion using enzymatic hydrolysis and synergistic cooperation between enzymes--factors affecting enzymes, conversion and synergy. Biotechnol Adv.

[CR50] Weiss N, Borjesson J, Pedersen LS, Meyer AS (2013). Enzymatic lignocellulose hydrolysis: improved cellulase productivity by insoluble solids recycling. Biotechnol Biofuels.

[CR51] Wigglesworth VB (1948). The structure and deposition of the cuticle in the adult mealworm, *Tenebrio molitor* L*.* (Coleoptera). Q J Microsc Sci.

