# Supplementary material for: High-throughput screening of environmental polysaccharide-degrading bacteria using biomass containment and complex insoluble substrates
Source: Appl Microbiol Biotechnol. 2020 Feb 29;104(8):3379–89. doi: 10.1007/s00253-020-10469-3 (PMC7089899; doi:10.1007/s00253-020-10469-3)
Supplement: Supplementary file 1 — (PDF 4590 kb) [file 253_2020_10469_MOESM1_ESM.pdf]

Applied Microbiology and Biotechnology

High-throughput screening of environmental polysaccharide-degrading bacteria using biomass containment and complex insoluble substrates

Estela C. Monge[1], Marios Levi[2], Joseph N. Forbin[1], Mussie D. Legesse[1], Basil A. Udo[1], Tagide N. deCarvalho[2], and Jeffrey G. Gardner[1#]

Running Title
High-throughput screening using biomass containment

Keywords
3D printing, *Cellvibrio japonicus*, cellulose, chitin, lignocellulose, polysaccharide

Author Affiliations
[1]Department of Biological Sciences, University of Maryland - Baltimore County
Baltimore, Maryland, USA
[2]Keith R. Porter Imaging Facility, University of Maryland - Baltimore County
Baltimore, Maryland, USA

[#]Correspondence
Jeffrey G. Gardner
Department of Biological Sciences
University of Maryland - Baltimore County
Email: jgardner@umbc.edu
Phone: 410-455-3613
Fax: 410-455-3875

Table S1 Strains used in this study

Strains	Relevant characteristics/Genotype	Source or Reference
*Cellvibrio japonicus Ueda*107	Prototroph (type strain)	NCIMB 10462
Cellvibrio japonicus Δ*gsp*	Ueda 107 Δ*gsp*	(Nelson and Gardner 2015)
Escherichia coli K-12 (MG1655)	F– λ^- *ilvG- rfb*-50 *rph*-1	NCIMB 10218
Arthrobacter nicotianae WW33[a]	Environmental sample isolated from leaf-cutting ant (*A. columbica)* colony	(Gardner et al. 2012)
Klebsiella oxytoca WW55[a]	Environmental sample isolated from wastewater treatment sludge	(Gardner et al. 2012)
Enterobacter env. clone WW64[a]	Environmental sample isolated from leaf-cutting ant (*A. cephalotes)* colony	(Gardner et al. 2012)
Bacillus firmus LZ66[a]	Environmental sample isolated from leaf-cutting ants (*A. columbica*) colony	(Gardner et al. 2012)
Arthrobacter nicotianae WW33[a]	Environmental sample isolated from leaf-cutting ant (*A. columbica)* colony	(Gardner et al. 2012)

Table S2 Growth statistics of *C. japonicus* mutants grown in defined medium for strains that correspond to Fig. 1e[b]

Device	Growth Rate[c]	Initial OD_{600}[d]	Max. OD_{600}[e]	T_1[f]	T_2[f]	Lag[g]
no device	0.30 ± 0.02	0.11 ± 0.01	1.06 ± 0.12	5	8	5
Mk-1m	0.20 ± 0.01	0.14 ± 0.03	1.04 ± 0.07	6	12	3
Mk-2m	0.20 ± 0.05	0.40 ± 0.13	1.31 ± 0.03	7	9	6
Mk-3m	0.29 ± 0.02	0.11 ± 0.01	1.16 ± 0.03	5	11	5

Table S3 Growth statistics of *C. japonicus* mutants grown in defined medium for strains that correspond to Fig. S6[b]

% of β-Chitin	Growth Rate[c]	Initial OD_{600}[d]	Max. OD_{600}[e]	T_1[f]	T_2[f]	Lag[g]
0.0%	ND[h]	0.09 ± 0.01	0.10 ± 0.02	ND	ND	ND
2.5%	0.06 ± 0.01	0.10 ± 0.01	0.64 ± 0.10	13	32	14
5.0%	0.08 ± 0.01	0.10 ± 0.01	0.68 ± 0.07	14	24	13
10.0%	0.09 ± 0.02	0.12 ± 0.01	0.92 ± 0.07	14	26	14
15.0%	0.11 ± 0.01	0.12 ± 0.02	1.18 ± 0.05	13	24	13

[a] Annotated by 16sRNA.
[b] Experiments were performed in biological triplicate; average and standard deviation reported.
[c] Growth rate is reported as generations per hour.
[d] Initial optical density (OD) at 600nm.
[e] Maximum growth as measured by optical density (OD) at 600nm.
[f] Time points used to calculate growth rate using previously reported methods [3] T_1 (initial) and T_2 (final).
[g] Lag phase was defined as the time required for a >25% increase in OD from the initial OD.
[h] Not Determined due to lack of growth.

Table S4 Growth statistics of *C. japonicus* mutants grown in defined medium for strains that correspond to Fig. 2[a]

Carbon source	Strain	Growth Rate[b]	Initial OD_{600}[c]	Max. OD[d]	T_1[e]	T_2[e]	Lag[f]
β-Chitin	*Cj* WT	0.22 ± 0.02	0.17 ± 0.03	1.67 ± 0.18	14	18	14
β-Chitin	*Cj* Δ*gsp*	ND[g]	0.11 ± 0.02	0.13 ± 0.03	ND	ND	ND
Glutinous rice	*Cj* WT	0.14 ± 0.01	0.16 ± 0.04	1.75 ± 0.01	10	18	8
Glutinous rice	*Cj* Δ*gsp*	ND	0.10± 0.01	0.11 ± 0.01	ND	ND	ND
Fungal biomass	*Cj* WT	0.19 ± 0.05	0.13 ± 0.03	1.03 ± 0.14	10	14	13
Fungal biomass	Cj Δ*gsp*	ND	0.15 ± 0.02	0.12 ± 0.01	ND	ND	ND
Mealworm cuticle	*Cj* WT	0.11 ± 0.01	0.27 ± 0.02	1.71 ± 0.18	8	12	5
Mealworm cuticle	*Cj* Δ*gsp*	ND	0.30± 0.13	0.63 ± 0.36	ND	ND	ND

Table S5 Growth statistics of *C. japonicus* mutants grown in defined medium for strains that correspond to Fig. 3[a]

Device	Carbon Source	Strain	Growth Rate[b]	Initial. OD[c]	Max. OD[d]	T1[e]	T2[e]	Lag[f]
mBCD	α-Chitin	*Cj* WT	0.02 ± 0.01	0.13 ± 0.02	0.89 ± 0.36	100	160	114
mBCD	α-Chitin	*Cj* Δ*gsp*	ND[g]	0.13 ± 0.02	0.14 ± 0.01	ND	ND	ND
mBCD	Filter paper	*Cj* WT	0.01 ± 0.01	0.12 ± 0.02	0.62 ± 0.15	40	100	26
mBCD	Filter paper	*Cj* Δ*gsp*	ND	0.10 ± 0.01	0.13 ± 0.01	ND	ND	ND
mBCD	Crab shell	*Cj* WT	0.03 ± 0.01	0.12 ± 0.01	0.56 ± 0.20	30	50	22
mBCD	Crab shell	*Cj* Δ*gsp*	ND	0.12 ± 0.02	0.20 ± 0.10	ND	ND	ND
BCD	α-Chitin	*Cj* WT	0.08 ± 0.02	0.02 ± 0.01	1.53 ± 0.15	72	95	72
BCD	α-Chitin	*Cj* Δ*gsp*	ND	0.03 ± 0.01	0.02 ± 0.01	ND	ND	ND
BCD	Filter paper	*Cj* WT	0.03 ± 0.01	0.04 ± 0.01	0.32 ± 0.04	24	48.5	24
BCD	Filter paper	*Cj* Δ*gsp*	ND	0.04 ± 0.02	0.04 ± 0.02	ND	ND	ND
BCD	Crab shell	*Cj* WT	0.11 ± 0.01	0.03 ± 0.01	0.62 ± 0.12	24	48.5	95
BCD	Crab shell	*Cj* Δ*gsp*	ND	0.02 ± 0.01	0.01 ± 0.01	ND	ND	ND

[a] Experiments were performed in biological triplicate; average and standard deviation reported.
[b] Growth rate is reported as generations per hour.
[c] Initial optical density (OD) at 600nm.
[d] Maximum growth as measured by optical density (OD) at 600nm.
[e] Time points used to calculate growth rate using previously reported method [3] T_1 (initial) and T_2 (final).
[f] Lag phase was defined as the time required for a >25% increase in OD from the initial OD.
[g] Not Determined due to lack of growth.

Table S6 Growth statistics for strains that correspond to Fig. S7[a]

Carbon Source	Strain[b]	Growth Rate[c]	Initial. OD_{600}[d]	Max. OD_{600}[e]	T_1[f]	T_2[f]	Lag[g]
Glucose	*Cj* WT	0.28 ± 0.01	0.12 ± 0	0.87 ± 0.01	5	9	5
Glucose	*Cj* Δ*gsp*	0.14 ± 0.01	0.12 ± 0	0.75 ± 0.02	4	14	5
Glucose	K-12	0.21 ± 0.01	0.13 ± 0.01	0.84 ± 0.14	7	14	7
Glucose	WW33	0.22 ± 0.02	0.13 ± 0	1.19 ± 0.01	9	17	9
Glucose	WW55	0.36 ± 0.01	0.13 ± 0.01	0.87 ± 0.01	2	7	3
Glucose	WW64	0.37 ± 0.04	0.13 ± 0	0.85 ± 0.85	3	6	4
Glucose	LZ66	0.21 ± 0.14	0.13 ± 0.01	0.36 ± 0.36	9	17	10

Table S7 Growth statistics for strains that correspond to Fig. 4[a]

Carbon Source	Strain[b]	Growth Rate[c]	Initial. OD_{600}[d]	Max. OD_{600}[e]	T_1[f]	T_2[f]	Lag[g]
Corn stover	*Cj* WT	0.02 ± 0.01	0.28 ± 0.03	0.98 ± 0.12	23	70	23
Corn stover	*Cj* Δ*gsp*	ND[h]	0.15 ± 0.02	0.16 ± 0.02	ND	ND	ND
Corn stover	K-12	ND	0.17 ± 0.03	0.21 ± 0.03	ND	ND	ND
Corn stover	WW33	0.02 ± 0.01	0.17 ± 0.03	0.98 ± 0.05	23	70	23
Corn stover	WW55	0.01 ± 0.01	0.17 ± 0.04	0.47 ± 0.09	47.5	118	23
Corn stover	WW64	0.01 ± 0.01	0.24 ± 0.06	0.69 ± 0.36	47.5	70	23
Corn stover	LZ66	0.02 ± 0.01	0.24 ± 0.03	0.86 ± 0.05	47.5	70	23
Glutinous rice	*Cj* WT	0.13 ± 0.02	0.16 ± 0.01	1.48 ± 0.11	10	20	8
Glutinous rice	*Cj* Δ*gsp*	ND	0.16 ± 0.03	0.22 ± 0.04	ND	ND	ND
Glutinous rice	K-12	ND	0.16 ± 0.02	0.19 ± 0.01	ND	ND	ND
Glutinous rice	WW33	0.25 ± 0.02	0.19 ± 0.02	1.83 ± 0.17	4	10	3
Glutinous rice	WW55	ND	0.18 ± 0.05	0.25 ± 0.08	ND	ND	ND
Glutinous rice	WW64	ND	0.19 ± 0.02	0.26 ± 0.26	ND	ND	ND
Glutinous rice	LZ66	0.24 ± 0.12	0.16 ± 0.04	1.01 ± 0.97	4	8	7

[a] Experiments were performed in biological triplicate; average and standard deviation reported.
[b] Tested strains: *Cellvibrio japonicus (Cj* WT*), Cellvibrio japonicus* Δ*gsp (Cj* Δ*gsp), Escherichia coli* K-12 (MG1655) (K-12), *Arthrobacter nicotianae* WW33 (WW33), *Klebsiella oxytoca* WW55 (WW55), *Enterobacter* env. clone WW64 (WW64*) and Bacillus firmus* LZ66 (LZ66).
[c] Growth rate is reported as generations per hour.
[d] Initial optical density (OD_{600}) at 600nm.
[e] Maximum growth as measured by optical density (OD) at 600nm.
[f] Time points used to calculate growth rate using previously reported method [3] T_1 (initial) and T_2 (final).
[g] Lag phase was defined as the time required for a >25% increase in OD from the initial OD.
[h] Not Determined due to lack of growth.

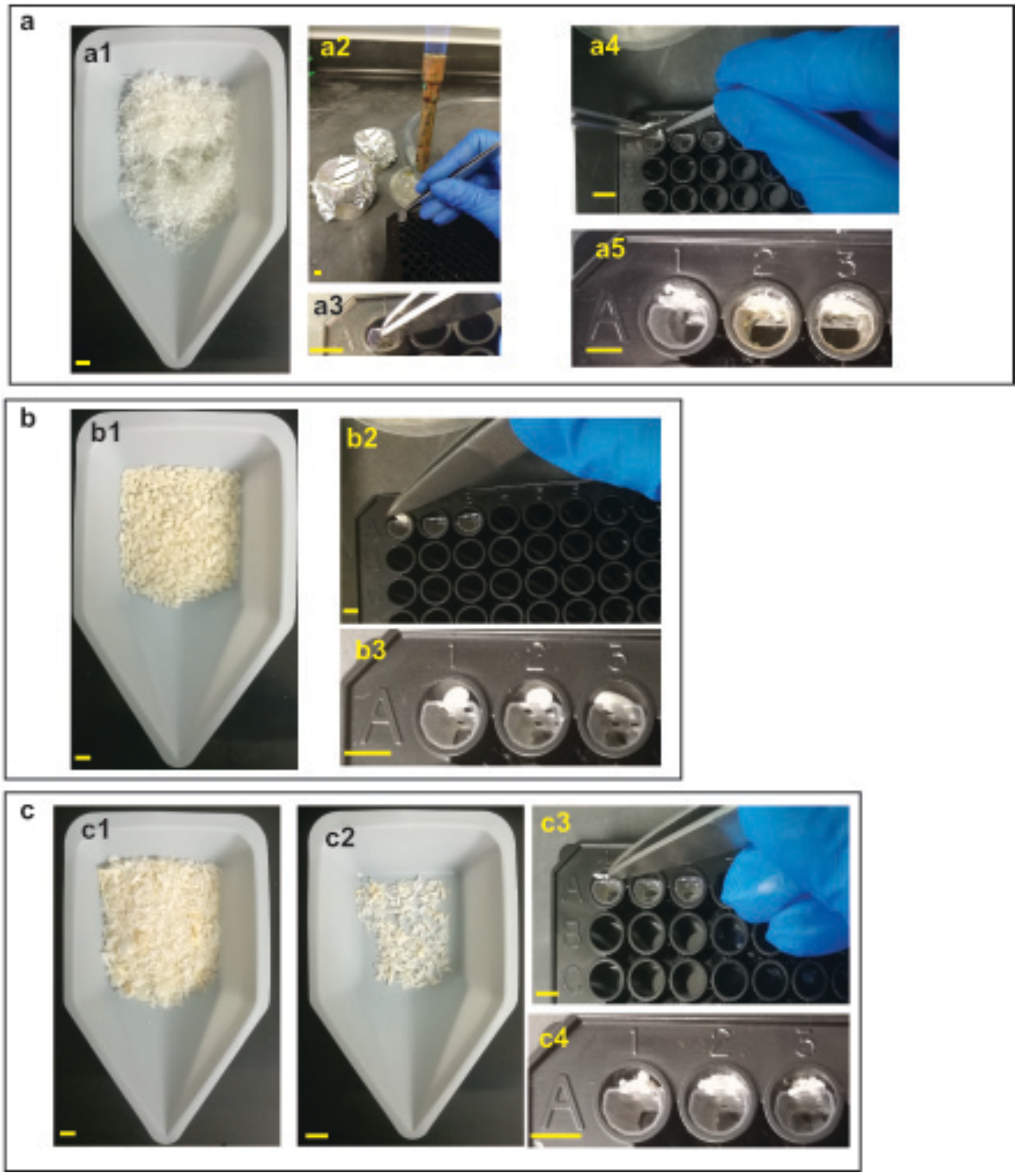

Fig. S1 Representation of the containment of β-chitin (a), glutinous rice (b) and α-chitin (c) in a microplate using Mk-3m devices. Autoclaved β-chitin fibers extracted from squid pen **(a1)** were aseptically transferred (**a2**) into a microplate containing an mBCD **(a3)** with a tweezers and a pipette tip **(a4)**. Approximately 10 mg of β-chitin were loaded into each well **(a5)**. Glutinous rice, washed, dried and autoclaved **(b1)** was transferred into a microplate **(b2)**. We loaded one grain per well **(b3)**. α-chitin flakes **(c1)** were sieved to select the ones that were 5 mm^2 **(c2).** The selected flakes were autoclaved and aseptically transferred with tweezers **(c3)**. We loaded 10 mg of α-chitin per well. The yellow bar under each image corresponds to 5 mm

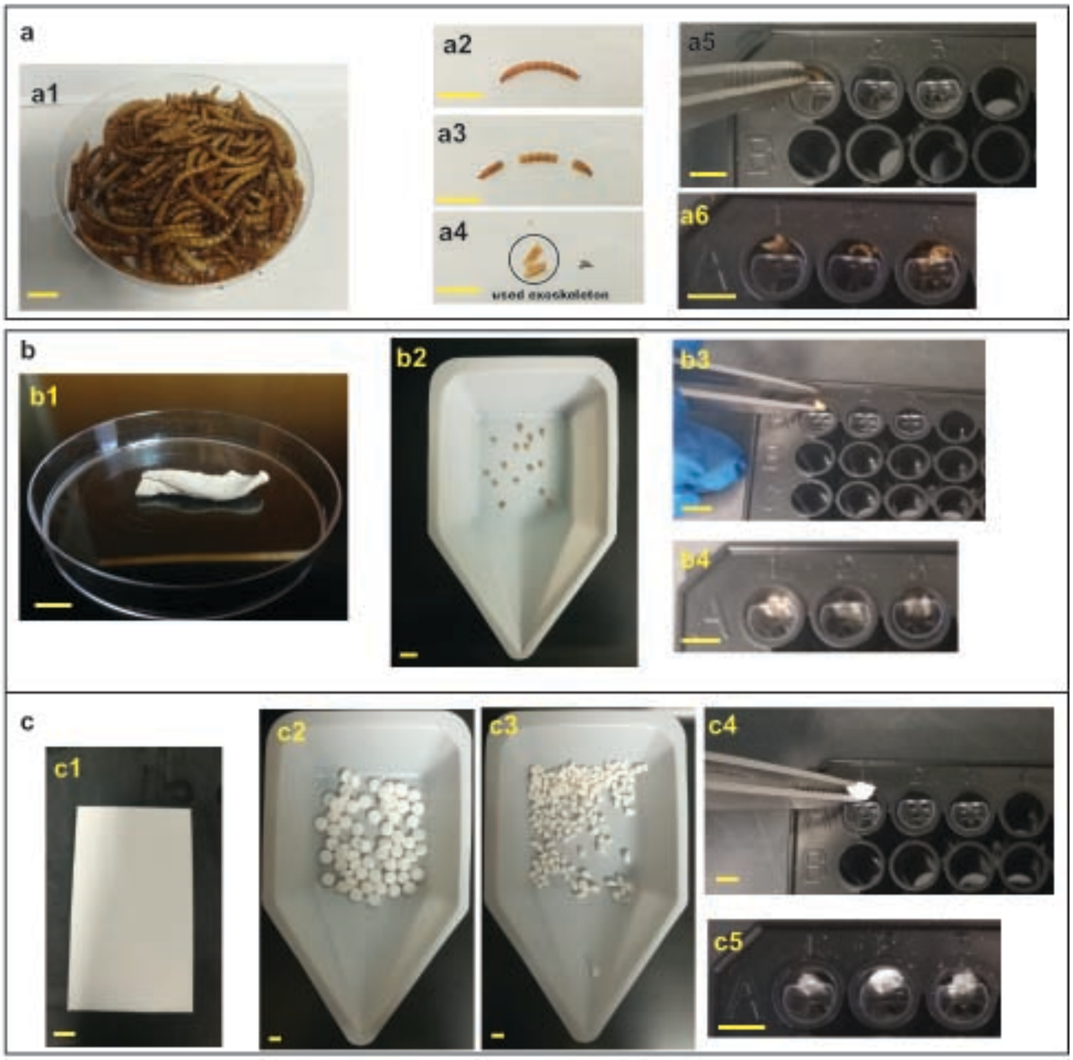

Fig. S2 Representation of the containment of mealworm cuticle (a), fungal biomass (b) and filter paper (c) in a microplate using Mk-3m devices. Larvae of the beetle *Tenebrio molitor* **(a1)** were dissected as follows: from each larva **(a2)** the thorax and the spine were removed **(a3)**. Then, the abdomen segment was split open to remove the intestines **(a4).** The selected exoskeleton was autoclaved and then aseptically transferred into a microplate with tweezers **(a5)**. Approximately 10 mg of mealworms were loaded into each well **(a6)**. Fungal biomass obtained from *Aspergillus nidulas* cultures **(b1)** was molded into 10 mg balls **(b2)**. Once molded, the biomass was autoclaved and transferred into a microplate **(b3)**. We loaded one ball per well **(b4)**. Whatman filter paper **(c1)** was punched**.** The collected punched dots **(c2)** were cut into quarters **(c3)** and autoclaved. Aseptically, the filter paper was transferred with tweezers into the microplate **(c4)**. We loaded 10 mg of filter paper per well **(c5).** The yellow bar under each image corresponds to 5mm

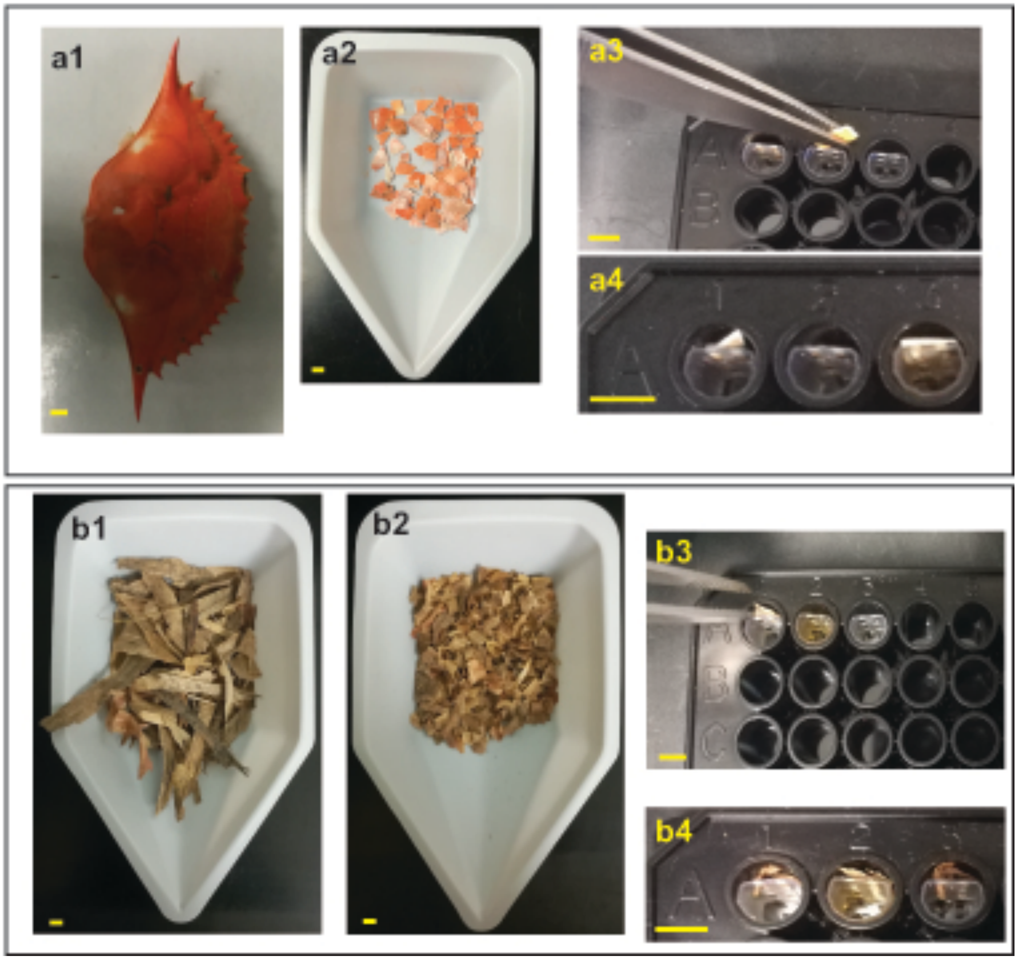

Fig. S3 Representation of the containment of crab shells (a) and corn stover (b) in a microplate using Mk-3m devices For the crab shells, the carapaces (**a1**) were selected and thoroughly washed and rinsed. Clean and air-dried carapaces were manually ground and then sieved through a 4 mm diameter filter. Only flat shells pieces were selected **(a2)**. To remove any generated crab shell dust, the filtrate was rinsed through a Buchner polypropylene filter. The shell pieces were subjected to autoclave sterilization as previously done (Monge et al. 2018) and then aseptically transferred into a microplate with tweezers **(a3)**. Approximately 20 mg of crab shells were loaded into each well **(a4)**. Stover from *Zea mays* **(b1)** was cut into pieces that were approximately 2.5 mm by 5 mm **(b2)**. The fragmented corn stover was thoroughly washed and autoclaved and then aseptically transferred to the microplate wells **(b3)**. We loaded 5 mg per plate well **(b4)**. The yellow bar under each image corresponds to 5 mm.

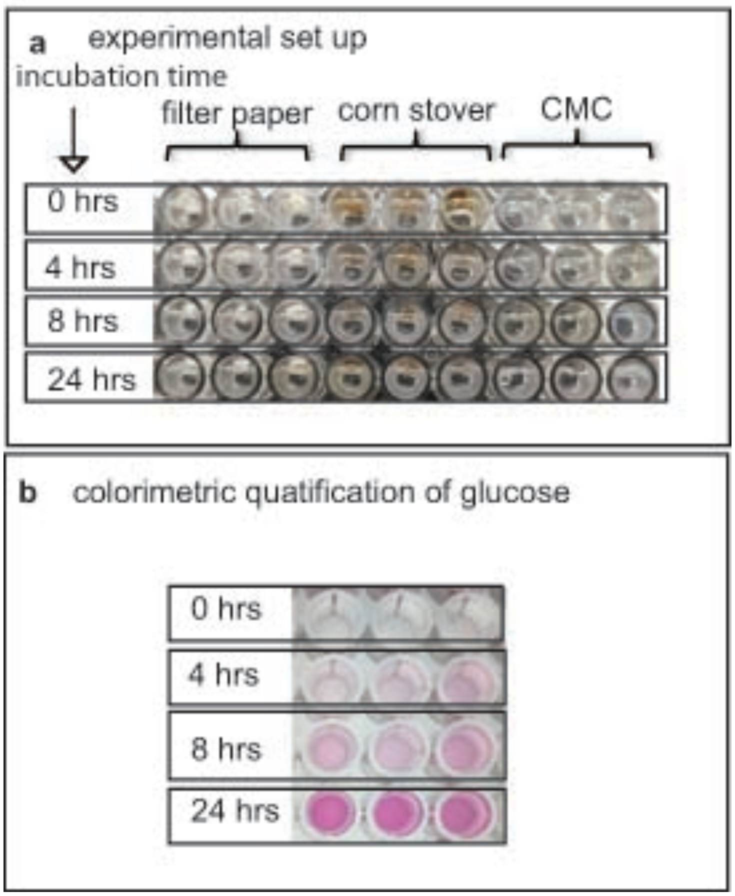

Fig. S4 Experimental set up for the enzymatic activity assays. For the enzymatic assays, filter paper and corn stover were incubated with the Cellulase for 0, 4, 8, and 24 hours and each sample was carried out in triplicate **(a).** The quantification of glucose was carried out in a microplate **(b)** and a calibration curve was employed to calculate the amount of relaxed glucose from filter paper and corn stover

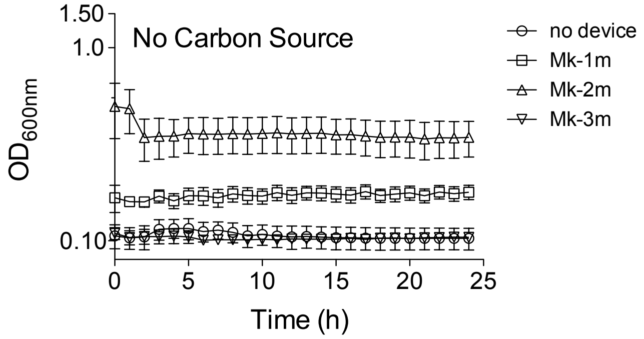

Fig. S5 Measurement of background generated from biomass containment using the Mk-1m, Mk-2m and Mk-3m devices. All experiments were done in biological triplicate at 30 °C. Error bars indicate standard deviation

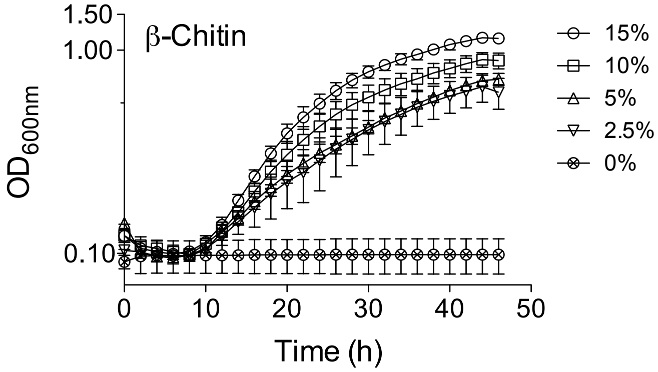

Fig. S6 Growth analysis of *C. japonicus* wild type on MOPS minimal media supplemented with different concentrations of β-chitin. All experiments were done in biological triplicate at 30 °C. Error bars indicate standard deviation. Purified chitin was the sole carbon source

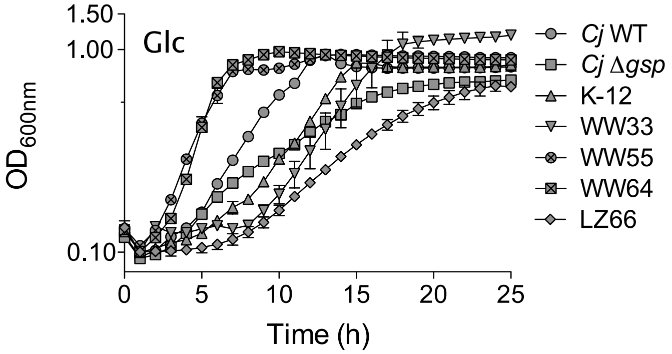

Fig. S7 Growth analysis using Mk-3m devices with environmental isolates. These were the tested strains *Cellvibrio japonicus (Cj* WT*), Cellvibrio japonicus Δgsp (Cj Δgsp), Escherichia coli* K-12, *Arthrobacter nicotianae* (WW33), *Klebsiella oxytoca* (WW55), *Enterobacter* spp. (WW64*) and Bacillus firmus* (LZ66). All growth analysis experiments were done in biological triplicate at 30 °C with high levels of aeration. Error bars indicate standard deviation. Glucose was the sole carbon source

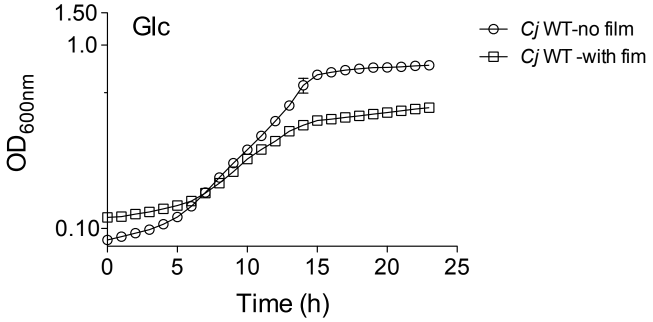

Fig. S8 Growth analysis of *Cellvibrio japonicus* growth using a microplate with and without a cover. The default protocol that used a cover plate (open circles) was compared to conditions where the cover plate was replaced with sealing film (open squares). All growth analysis experiments were done in biological triplicate at 30 °C with high levels of aeration using glucose as the sole carbon source. Error bars indicate standard deviation

REFERENCES

Gardner JG, Zeitler LA, Wigstrom WJ, Engel KC, Keating DH (2012) A high-throughput solid phase screening method for identification of lignocellulose-degrading bacteria from environmental isolates. Biotechnol Lett 34(1):81-89

Monge EC, Tuveng TR, Vaaje-Kolstad G, Eijsink VGH, Gardner JG (2018) Systems analysis of the family glycoside hydrolase family 18 enzymes from *Cellvibrio japonicus* characterizes essential chitin degradation functions. J Biol Chem 293(10):3849-3859

Nelson CE, Gardner JG (2015) In-frame deletions allow functional characterization of complex cellulose degradation phenotypes in *Cellvibrio japonicus*. Appl Environ Microbiol 81(17):5968-5975
